# The retained-bridge traction strategy: a novel solution for secure defect closure following duodenal endoscopic full-thickness resection

**DOI:** 10.1055/a-2804-6504

**Published:** 2026-03-09

**Authors:** Keyang Zhang, Shuqian Hu, Xueting Zhang, Min Min, Yan Liu

**Affiliations:** 1 12538 The School of Medicine, Nankai University, Tianjin, China; 2 Department of Gastroenterology, The First Medical Center of PLA General Hospital, Beijing, China

Endoscopic full-thickness resection (EFTR) in the duodenum presents unique challenges attributing to its “C-loop” configuration, narrow lumen, thin muscle layer and abundant vascularity 1 . Furthermore, defect closure is particularly challenging when lesions are located at sharply angulated segments of the duodenal bulb, such as the posterior or posterosuperior wall, where the unstable endoscope position and non-colinear alignment of the resection margins frequently impede reliable edge-to-edge approximation 2 . Consequently, a simple and effective strategy to facilitate defect alignment and secure closure in these anatomically unfavorable locations remains an unmet clinical need.

We present a novel retained-bridge traction strategy for secure defect closure following duodenal EFTR ( Video 1 ).

The retained-bridge traction strategy: a novel solution for secure defect closure following duodenal endoscopic full-thickness resection.Video 1

**Fig. 1:**
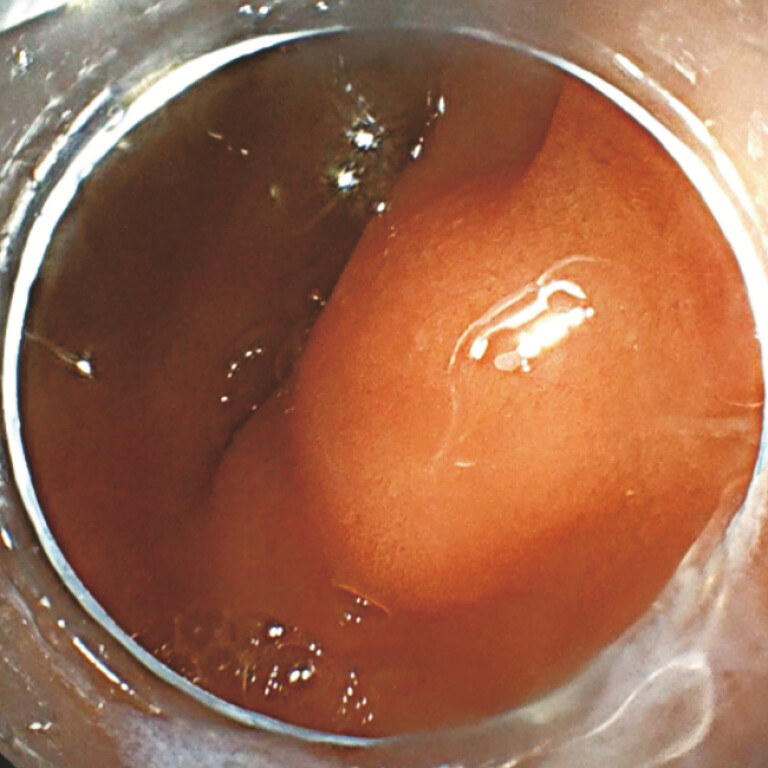
Subepithelial lesion in the duodenal bulb.

**Fig. 2:**
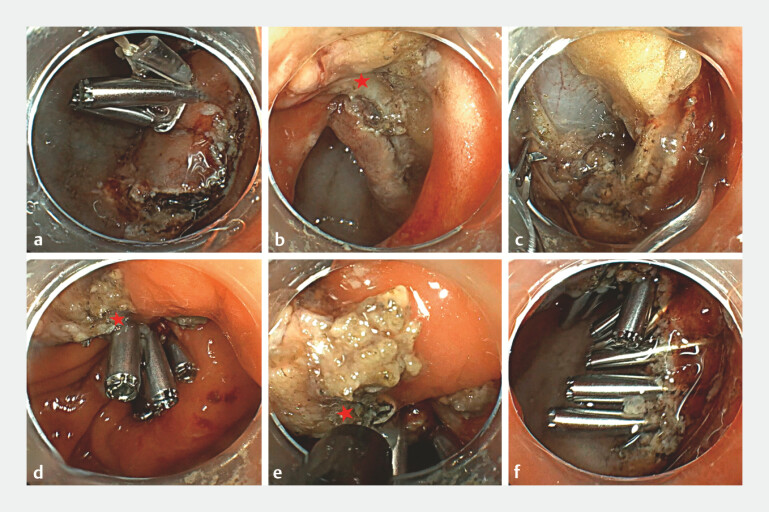
**a** A traction device: a dental floss loop anchored by two clips. **b** A tissue bridge is intentionally preserved at the margin. Under traction, the defect is flattened with edges approximated. **c** Novel two-tooth clips (TTC) are deployed along the ridge. **d** The preserved tissue is securely clamped with the TTC. **e** The preserved tissue is transected using an electrosurgical knife. **f** Complete closure without leakage or luminal stenosis. The red star indicates the preserved tissue bridge.

A 43-year-old man was admitted for a subepithelial lesion in the duodenal bulb ( Fig. 1 ), suspected to be a gastrointestinal stromal tumor on endoscopic ultrasound. EFTR was performed using a traction device consisting of a dental floss loop anchored by two clips ( Fig. 2 **a** ). After completing a sub-circumferential full-thickness incision, the lesion was intentionally not completely detached unlike the intermittent “cut-and-sew” technique. Instead, a small tissue bridge was preserved at the lesion margin ( Fig. 2 **b** ). This retained tissue bridge functioned as a mechanical anchor, providing continuous counter-traction that transformed the irregular and unstable defect into a well-aligned linear ridge. Novel high-force clips were then sequentially deployed along this ridge to achieve secure full-thickness closure ( Fig. 2 **c** 3 ). Subsequently, the preserved tissue bridge was firmly clamped ( Fig. 2 **d** ) and transected using an electrosurgical knife ( Fig. 2 **e** ). Final endoscopic inspection confirmed the complete defect closure without leakage or luminal stenosis ( Fig. 2 **f** ). The patient resumed diet on day 3 and was discharged on day 4 without any adverse event. Histopathology confirmed ectopic pancreas tissue (1.5 × 1.5 × 0.8 cm).

In conclusion, the retained-bridge traction strategy effectively addresses one of the key technical limitations of duodenal EFTR, particularly in sharply angulated posterior bulb lesions. By converting an unstable defect into a stable and well-aligned ridge, this technique facilitates reliable closure and may reduce the risk of perforation and delayed bleeding in high-risk duodenal locations.

Endoscopy_UCTN_Code_TTT_1AO_2AG_3AF
